# Supplementary material for: Association between oral microbiome and seven types of cancers in East Asian population: a two-sample Mendelian randomization analysis
Source: Front Mol Biosci. 2023 Nov 21;10:1327893. doi: 10.3389/fmolb.2023.1327893 (PMC10702768; doi:10.3389/fmolb.2023.1327893)
Supplement: Supplementary file 1 [file DataSheet1.ZIP › Supplementary Materials/MR plots of saliva/saliva═╝/Prostate cancer/pheno.3408_to_prostate cancer_forest.pdf]

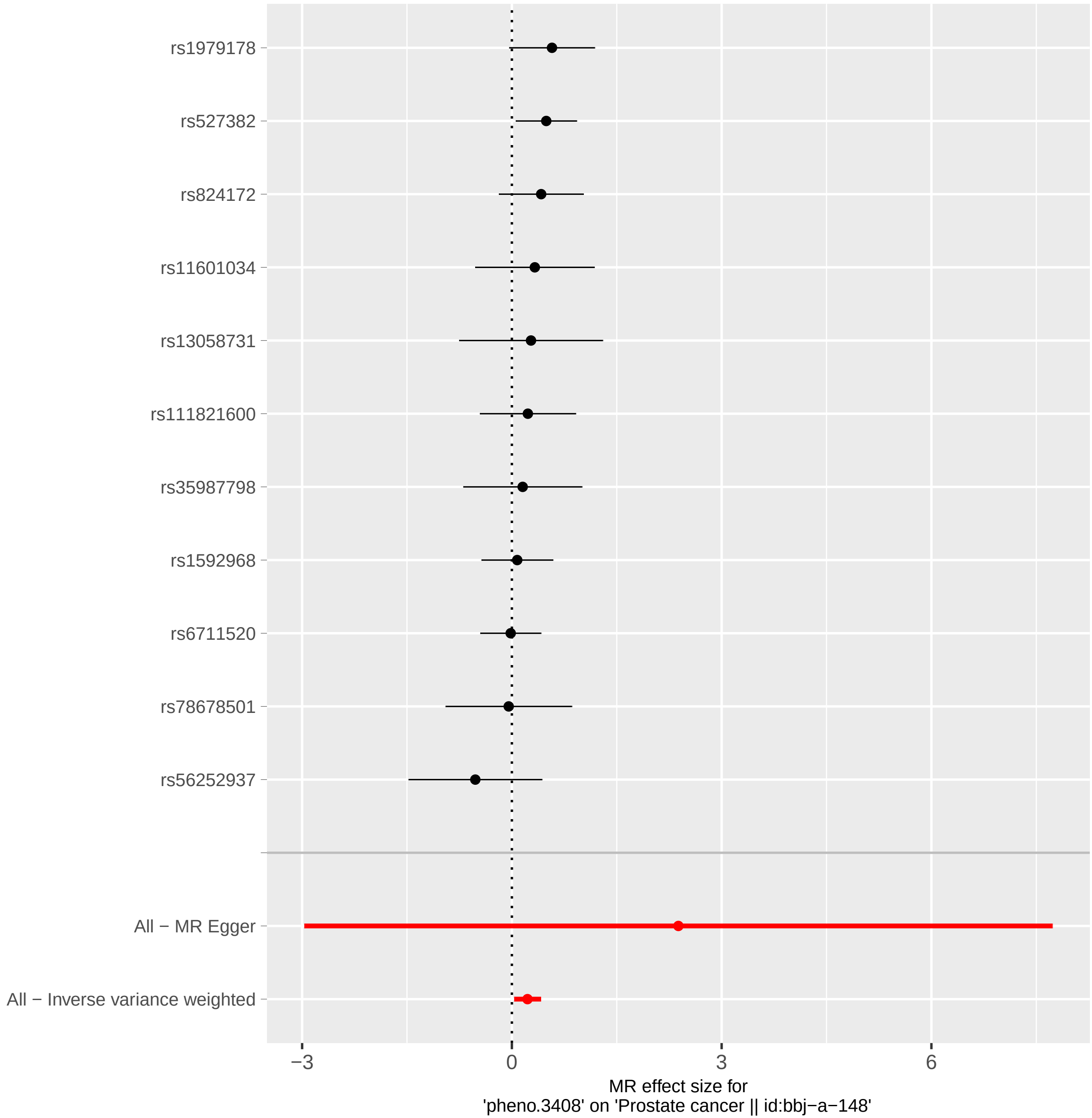
rs1979178
rs527382
rs824172
rs11601034
rs13058731
rs111821600
rs35987798
rs1592968
rs6711520
rs78678501
rs56252937
All − MR Egger
All − Inverse variance weighted
−3
0
3
6
MR effect size for
'pheno.3408' on 'Prostate cancer || id:bbj−a−148'
